# To see or not to see (again): Dealbreakers and dealmakers in relation to social inclusion

**DOI:** 10.3389/fpsyg.2022.1019272

**Published:** 2022-09-23

**Authors:** Peter K. Jonason, Kaitlyn P. White, Abigail H. Lowder, Laith Al-Shawaf

**Affiliations:** ^1^Department of General Psychology, University of Padua, Padua, Italy; ^2^Institute of Psychology, University of Cardinal Stefan Wyszyński, Warszawa, Poland; ^3^Department of Psychology, Pikes Peak College, Colorado Springs, CO, United States; ^4^Neuroscience Program, Hamilton College, Clinton, NY, United States; ^5^Department of Psychology, University of Colorado, Colorado Springs, CO, United States

**Keywords:** mate choice, decision making, interpersonal relationships, mate value, courtship (dating)

## Abstract

In this study, we replicated what is known about the relative importance of dealbreakers (i.e., traits avoided) and dealmakers (i.e., traits sought) in romantic and sexual relationships and extended it to an examination of self-reports of mate value, self-esteem, and loneliness. In two experiments (*N* = 306; *N* = 304) we manipulated the information people were told about potential partners and asked them about their intentions to have sex again with or go on a second date with opposite sex targets. People were less interested in partners after learning dealbreakers, effects which operated more strongly in the long-term than short-term context, but similarly in men and women. People who reported less self-esteem or more loneliness were more receptive to people with dealbreakers. People who thought they had more mate value, more self-esteem, or less loneliness were more receptive to dealmakers. Results are discussed using sociometer, prospect, and sexual strategies theories.

## Introduction

In the initial stages of selecting a romantic or sexual partner, people engage in two evaluative processes. The first of these is to eliminate those partners that have problematic features like being unhygienic, aggressive, and likely to have sexually transmitted infections—so-called *dealbreakers* (Jonason et al., 2015; Csajbók and Berkics, 2017; Stewart-Williams et al., 2017; Apostolou and Eleftheriou, 2022). This process is about minimizing time lost pursuing and the potential costs associated with dating low quality or unsuitable partners. The second process is where individuals select among those who have sufficient quality—passing minimum thresholds (Li et al., 2002, 2013)—based on their desirable qualities like being physically attractive, funny, and kind—so-called *dealmakers* (Buss, 1989; Kenrick et al., 1990; Machia and Ogolsky, 2020).

However, most research on these topics (1) operates in isolation, not considering both aspects of mate choice, (2) typically relies on normative responses (e.g., willingness) instead of ipsative (e.g., yes/no) responses or change in interest which both may better resemble mate choice as opposed to mate preferences, and (3) attempts to understand the role of a single (or a small number of) indicators of mate choice instead of a holistic dealbreaker/dealmaker index. Moreover, this research has focused on a limited range and haphazard assortment of individual differences to account for dealbreaker/maker effects like mate value, sociosexuality, the Dark Triad traits, and disgust mechanisms. Therefore, in this study, we attempt to replicate what is known about the importance of dealbreakers and dealmakers in mate choice overall and in men and women along with the role of three traits that we think capture different aspects of people’s sense of social inclusion (i.e., mate value, self-esteem, and loneliness).

### Sex differences in mate choice

One of the most fundamental assertions in evolutionary models of mating psychology suggests that the degree to which men and women differ in aspects of their mate preferences and willingness to have casual sex originates in the past (Buss and Schmitt, 1993). Ancestral women who were more promiscuous would have had limited resources (e.g., time conflicts between rearing and food acquisition; Trivers, 1972) and lack of help from the father of her child would have undermined her and her offspring’s reproductive fitness. This would have selected women who were more risk averse in their mating psychology than men are (as is the case in most mammals), which will translate to them reacting more adversely to dealbreakers today (White et al., 2020; Jonason et al., 2020b) to mitigate the associated risks (Haselton and Buss, 2000; Saad et al., 2009; Saad and Gill, 2014).

In contrast, ancestral men would have derived greater benefit from casual encounters because they have less obligatory investment in any sexual encounter (Trivers, 1972). This translates into men being more tuned towards dealmakers to maximize their reproductive fitness. Stated another way, women may be looking for reasons to say “no” whereas men may be looking for reasons to say “yes”, especially with respect to short-term mates. However, in the context of long-term relationships, both sexes have relatively high investment in making good mating decisions (Buss and Schmitt, 1993; Li et al., 2002, 2013), and thus sex differences in the sensitivities to dealbreakers and dealmakers should be similar in nature.

### Individual differences in real or perceived social inclusion

While sex differences are interesting, they fail to provide information about the psychological mechanisms that may be associated with responses to different classes of information about prospective partners. We contend that people’s sense of social inclusion may influence their responses in adaptive or maladaptive ways. We base our thinking loosely on the sociometer theory that suggests that measure of one’s sense of social inclusion are outcomes based on feedback from the world in terms of how much others like them and want to affiliate with them (Leary et al., 1995; Leary, 1999; Kirkpatrick and Ellis, 2001). Social inclusion may come in at least three forms: mate value (i.e., a person’s sense of embodying qualities that prospective partners are interested in; Waynforth, 2001; Kirsner et al., 2003; Millar et al., 2019), self-esteem (i.e., their sense of general acceptance by others; Leary, 1999), and loneliness (i.e., the emotion of feeling left out, unsupported, not belonging; Russell, 1996).

People who have higher mate value may think they are better suited to find romantic partners in the future and, therefore, are more likely to lose interest in potential partners who are insufficient in their eyes (Jonason et al., 2015, 2020a). Like mate value, self-esteem (Brase, 2004; Schmitt and Jonason, 2019) may track how people feel they are valued by others (Leary et al., 1995; Erol and Orth, 2016) and thus lead people to reject others characterized by dealbreakers and accept those characterized by dealmakers to a greater extent. And last, loneliness may be especially unique in terms of social inclusion and dating. Feelings of loneliness are higher among people who lack a romantic partner (Adamczyk, 2016) but it is unclear if this is because they reject too many partners or fail to attract others. In fact, we propose that loneliness, as a dispositional trait, may create a self-perpetuating system that maintains a homeostasis in loneliness. The fact that loneliness should be solvable—meet more people, be more social, go on more dates—may suggest that chronically lonely people mistakenly over-accept lower quality mates (i.e., those with dealbreakers) and over-reject higher quality mates (i.e., those with dealmakers).

### The current studies

In two studies we attempt to methodologically improve on and replicate previous research on men and women’s responses to learning dealbreaker and dealmaker information about potential sexual and romantic partners. We extend this to include a broader assessment of the role of social inclusion in predicting responses to this information with a more inclusive measure of mate value (e.g., not focused solely on long-term mate value; Kirsner et al., 2003) and measures of self-esteem and loneliness. We expect each to reveal unique decision-making biases in relation to dealbreaker/maker information. For instance, those with high mate value may be focused on dealmakers because they perceive themselves as having high value, thus focusing them on the upsides of dating whereas those who feel lonely may be so because they are too inclined to reject others characterized by dealmakers but because they are lonely, they may simultaneously fail to reject people who are characterized by dealbreakers.

## Materials and methods

### Participants and procedures

Two online studies drew on Amazon Mechanical Turk workers from the United States in 2020 who were paid US$0.50 or US$1.00, respectively. Participants from Study 1 (*N* = 306; 179 men) were aged between 18 and 65 (*M* = 35.76, *SD* = 10.22), mostly heterosexual (76%), and in a committed relationship (81%).^1^ Participants from Study 2 (*N* = 304; 134 men) were aged between 18 and 65 (*M =* 33.09*, SD =* 10.28), mostly heterosexual (83%), and in a committed relationship (72%).^2^ We attempted (and fell a little short) to collect double the minimum sample size (*N_one-tailed_* ≈ 175) to detect a moderate effect size (Cohen’s *d* ≈ −0.38; Jonason et al., 2020b) to best assess correlational effects. Participants were provided the details of the study and provided click-to-continue consent. They were then randomly assigned to either a dealbreaker (e.g., “Smells bad,” “Has a sexually transmitted infection”) or dealmaker (e.g., “Is well educated,” “Is kind to strangers”) condition before completing the scales (items randomized) detailed below. Upon completion, participants were thanked and debriefed. These studies were approved by the ethics committee at Western Sydney University (H14099) and Putnam Valley High School (72021) and the data (not pre-registered) is available on the Open Science Framework.^3^

### Measures

We devised two methods to capture change in interest as a function of learning dealbreaker/maker information about potential long-term or short-term partners (within-subjects in both cases). In Study 1 participants were instructed to imagine they had met someone who they liked and found physically attractive (establishing initial interest) before being asked to read a list of randomized characteristics (Jonason et al., 2020b). In Study 1, participants were randomly assigned to either the dealbreaker or dealmaker condition and asked how learning this information would affect their interest in the target (−3 = *Greatly decrease interest*; +3 = *Greatly increase interest*). The items in each condition were consistent between participant and participants responded to each dealbreaker or dealmaker item individually. We averaged 10 items to measure individual differences in responses to dealmakers (Cronbach’s *α* = 0.90) and 10 items to measure individual differences in dealbreakers (*α* = 0.98). In Study 2, we used the same 20 items^4^ but now adopted a within-subjects design and, instead, participants were asked to imagine they had already had sex with or gone on a date with a target (thereby establishing initial attraction) and whether they would have sex again or go on another date with that person (yes/no) after learning the new information. The number of “yes” responses for *sex* in response to learning dealbreakers (*ɑ* = 0.77) and dealmakers (*ɑ* = 0.77) and the number of “yes” responses for another *date* in response to learning dealbreakers (*ɑ* = 0.74) and dealmakers (*ɑ* = 0.66) were totaled.

In Study 1, we used a 20-item mate value scale (Jonason et al., 2019, 2020a) to assess three aspects of self-reported mate value. Participants rated their agreement (1 = *strongly disagree*; 7 = *strongly agre*e) with items like “Compared to my peers, I am very attractive or desirable to the opposite sex” (i.e., short-term attractiveness), “Characteristics of mine other than physical attractiveness seem to appeal to potential romantic partners” (i.e., long-term attractiveness), and “I tend to have a more difficult time attracting potential mates than other people do” (i.e., general unattractiveness). Because we failed to find much differentiation in our results on the three kinds of mate value (which were moderately correlated^5^), we averaged items across the three measures of mate value to capture individual differences in overall mate value (*α* = 0.82).

In Study 2, we used the 20-item UCLA Loneliness Scale (Russell et al., 1978) to assess loneliness. Participants rated their agreement (1 = *Strongly Agree*; 5 = *Strongly Disagree*) with each item. Items included questions such as “I lack companionship.” We averaged the items to form a composite score of loneliness (*ɑ =* 0.94).

In Study 2, we used the 10-item Self Esteem Scale (Rosenberg, 1965) to measure the self-esteem of each participant. Participants rated their agreement (1 = *Strongly Disagree*; 5 = *Strongly Agree*) with items such as “I take a positive attitude toward myself.” We averaged items to form a composite score of self-esteem (*ɑ = 0*.87).

### Results

To begin, we tested a 2 (information learned: dealbreaker vs. dealmaker) × 2 (participant’s sex) × 2 (mating context: short-term vs. long-term) mixed model ANOVA (see Table 1) in the Study 1 data to replicated sex, context, and informational effects. Overall, participants (regardless of their sex) who learned dealbreakers (*M* = −0.36*, SD* = 1.76)^6^ were less interested (*F* [1, 302] = 182.87, *p* < 0.001, *η_p_*^2^ = 0.38) in a potential partner than participants who learned dealmakers (*M* = 1.67, *SD* = 0.69). We found an interaction between information learned and participant’s sex (*F* [1, 302] = 4.51, *p* = 0.035, *η_p_*^2^ = 0.02), suggesting that, overall, after learning dealbreakers, women were less interested in a potential partner than men were, but were more interested than men after learning dealmakers. Within both men (*t* = −8.94, *p* < 0.001) and women (*t* = −10.09, *p* < 0.001), those who learned dealbreakers were less interested in a prospective partner. However, planned comparisons revealed no overall difference between men and women in change in interest after learning dealbreakers or dealmakers. We also found an interaction between mating context and information learned (*F* [1, 302] = 12.16, *p* = 0.001, *η_p_*^2^ = 0.04; see Table 1). Further analysis found that people who learned dealmakers about a person were more interested in that person as a long-term partner than as a short-term partner (*t* = 2.90, *p* = 0.004). When looking at men and women separately, this effect only held among women (*t* = 3.61, *p* = 0.001).

**Table 1:** Between- and within-subjects effects of participants’ sex and the presentation of dealbreakers or dealmakers on change in interest in short-term and long-term mating contexts (Study 1).

	Mean (*SD*)			*t*-test	Cohen’s *d*
	Overall	Men	Women		
**Short-term mating context**	0.64 (1.66)	0.72 (1.57)	0.53 (1.79)	0.96	0.11
Types of information					
Dealbreakers	−0.32 (1.76)	−0.13 (1.71)	−0.58 (1.81)	1.58	0.26
Dealmakers	1.60 (0.77)	1.56 (0.78)	1.66 (0.75)	−0.81	−0.13
*t*-test	−12.35**	−8.47**	−9.14**		
Cohen’s *d*	−1.41	−1.27	−1.62		
**Long-term mating context**	0.67 (1.73)	0.73 (1.62)	0.58 (1.88)	0.71	0.09
Types of information					
Dealbreakers	−0.40 (1.79)	−0.19 (1.74)	−0.69 (1.84)	1.74	0.28
Dealmakers	1.73 (0.72)	1.63 (0.77)	1.88 (0.61)	−2.18*	−0.36
*t*-test	−13.65**	−9.04**	−10.60**		
Cohen’s *d*	−1.56	−1.35	−1.87		

*p < 0.05; **p < 0.01.

Second, we examined whether people’s interest in mates with dealmakers and dealbreakers was related to their sense of social inclusion (see Table 2) in the data from both studies. Mate value was unrelated to change in interest in response to dealbreakers in either sex, but it was more sensitive to dealmakers. Men’s mate value was less sensitive to dealmakers in the long-term condition compared to both women and the short-term condition. In contrast (albeit with a different method), self-esteem was more sensitive to both dealbreaker and dealmaker information with positive correlations in men (but not women) in response to dealbreakers with the same pattern emerging for self-esteem as mate value in response to dealmakers. And last, loneliness was especially sensitive to dealbreaker and dealmaker responses with lonelier people saying “no” to dealbreakers *less* and “no” to dealmakers *more*.

**Table 2:** Correlations for self-perceived mate value, self-esteem, and loneliness and interest with mates with dealmakers and dealbreakers in short-term and long-term mating contexts and within men and women (Studies 1 and 2).

Mate value	Overall	Men	Women	Fisher’s *z*
Dealbreakers				
Long-term	−0.12	−0.03	−0.20	1.01
Short-term	−0.10	−0.02	−0.16	0.83
Steiger’s *z*	−1.01	0.38	0.88	
Dealmakers				
Long-term	0.31**	0.18	0.47**	−2.16*
Short-term	0.38**	0.36**	0.39**	−0.20
Steiger’s *z*	−1.21	−2.29*	1.36	
**Self-esteem**				
Dealbreakers				
Long-term	0.17**	0.24**	0.12	1.07
Short-term	0.15**	0.18*	0.14	0.42
Steiger’s *z*	0.60	0.87	−0.31	
Dealmakers				
Long-term	−0.17**	−0.13	−0.22**	0.79
Short-term	−0.20**	−0.22*	−0.20**	−0.18
Steiger’s *z*	0.59	1.06	−0.37	
**Loneliness**				
Dealbreakers				
Long-term	−0.31**	−0.31**	−0.31**	0.07
Short-term	−0.30**	−0.21*	−0.37**	1.48
Steiger’s *z*	0.41	−0.03	−1.44	
Dealmakers				
Long-term	0.20*	0.20*	0.20**	−0.27
Short-term	0.19**	0.26**	0.14	1.06
Steiger’s *z*	−0.12	0.79	−0.97	

*p < 0.05; **p < 0.01.

Third, we tested a 2 (information learned: dealbreaker vs. dealmaker) × 2 (participant’s sex) × 2 (mating context: short vs. long-term) mixed model ANOVA (see Table 3) for the Study 2 data. Overall, after learning dealbreakers (*M* = 4.27, *SD* = 4.14)^7^ participants (regardless of their sex) were less interested (*F* [1, 302] = 1701.15, *p* < 0.001, *η_p_*^2^ = 0.85) in a potential partner than after learning dealmakers (*M* = 17.36, *SD* = 3.11). Additionally, men (*M* = 22.26, *SD* = 4.71) indicated an interest in a second encounter with the prospective mate more frequently (regardless of the information learned; *F* [1, 302] = 4.23, *p* = 0.041, *η_p_*^2^ = 0.01) than women (*M* = 21.13, *SD* = 4.81). Moreover, we found an interaction between information learned and participants’ sex (*F* [1, 302] = 10.64, *p* = 0.001, *η_p_*^2^ = 0.03), suggesting that men were more likely than women to say “yes” to potential partners after learning dealbreakers; an effect that was present in both short-term (*t =* −3.44*, p* < 0.001) and long-term contexts (*t =* −2.81*, p* = 0.005). Additionally, we found an interaction between mating context and information learned (*F* [1, 302] = 10.94, *p* = 0.003, *η_p_*^2^ = 0.03) such that, after learning dealbreakers about a person, people responded “no” *more* frequently in the long-term context than in the short-term context. On the other hand, after learning dealmakers about a person, people responded “no” *less* frequently in the long-term context than in the short-term context (see Table 3). Further analysis found that after learning dealmakers about a person led to more interest that person as a long-term partner than as a short-term partner (*t* = 2.53, *p* = 0.012); an effect localized to women (*t* = 2.18, *p* = 0.031).

**Table 3:** Between- and within-subjects effects of participants’ sex and the presentation of dealbreakers or dealmakers on change in interest in short-term and long-term mating contexts (Study 2).

	Mean (*SD*)			*t*-test	Cohen’s *d*
	Overall	Men	Women		
	10.78 (2.84)	11.16 (2.88)	10.48 (2.77)	−2.09*	−0.24
Types of information					
Dealbreakers	2.21 (2.27)	2.71 (2.35)	1.81 (2.13)	−3.44**	−0.40
Dealmakers	8.57 (1.92)	8.45 (1.88)	8.66 (1.95)	−0.98	−0.11
*t*-test	−35.73**	−21.17**	−29.82**		
Cohen’s *d*	−3.03	−2.70	−3.35		
Long-term mating context	10.85 (2.38)	11.10 (2.44)	10.65 (2.31)	−1.65	0.19
Types of information					
Dealbreakers	2.06 (2.13)	2.45 (2.27)	1.75 (1.96)	−2.81 **	−0.33
Dealmakers	8.79 (1.53)	8.66 (1.66)	8.90 (1.41)	−1.38	−0.16
*t*-test	−41.36**	−22.95**	−37.01**		
Cohen’s *d*	−3.63	−3.12	−4.19		

*p < 0.05; **p < 0.01.

## Discussion

In the current studies, we replicated previous research showing dealbreakers to be less appealing than dealmakers, especially in the long term (compared to short term) context and to women (compared to men). Men’s mate value was less sensitive to dealmakers than women and in the long-term compared to the short-term context, whereas men’s self-esteem was more sensitive than women’s to both dealbreakers and dealmakers. Loneliness was also sensitive to dealbreaker and dealmaker information, with lonelier people reporting greater openness to partners with dealbreakers and lower openness to partners with dealmakers.

There are few decisions in people’s lives more important than choosing a mate. Such decisions have implications for both psychological health and evolutionary fitness. Choosing who to pursue and who to avoid are two different mate selection processes that serve to avoid the costs of mating mistakes and then maximizing the benefits people may accrue from engaging with a particular partner; these are not symmetrical, and they are not symmetrically evaluated in people (Buss, 1989; Buss and Schmitt, 1993;Haselton and Buss, 2000; Jonason et al., 2015). Prior research has predominantly focused on the traits people seek, but both processes are important to consider. When research has considered both, it may not have captured the degree to which new information leads to changes in interest in potential partners—instead focusing on how much a trait could be considered a dealbreaker or the factor structure of those traits (Jonason et al., 2015; Csajbók and Berkics, 2017).Moreover, attempts to understand individual differences in responses to dealbreaker/maker information has been rather haphazard. Therefore, in two experiments we examined how learning dealbreaker and dealmaker information changed—in a graded or a dichotomous way—people’s interest in continuing a romantic or sexual relationship with a prospective mate with whom they are (hypothetically) already attracted to and interested in, and how these responses are moderated by sex and related to individual differences in one’s sense of social inclusion.

Primarily we replicated prior research on dealbreakers (Jonason et al., 2015, 2020b; White et al., 2020). Our findings suggest that men and women are less interested in prospective mates after learning dealbreakers compared to dealmakers, although this was stronger in women than in men and in the long-term than the short-term contexts. This suggests that while both sexes may want to avoid partners characterized by undesirable traits, women especially may seek to avoid mating mistakes more than men (Haselton and Buss, 2000; Jonason et al., 2015). In addition, people are more interested in a mate with dealbreakers as a short-term, rather than long-term, partner and are more interested in a mate with dealmakers as a long-term, rather than short-term, partner. This suggests that people try to avoid mating mistakes more in the short-term context but seek mating benefits more in the long-term context. Mating mistakes can have serious reputational and health consequences and those may be highest in casual sex partners given the limited commitment between the partners.

We also tried to understand the role of social inclusion in accounting for people’s responses to learning dealbreaker/maker information. We expected social inclusion to be multi-faceted and, thus, we assessed it in the context of mating (i.e., mate value; Waynforth, 2001; Kirsner et al., 2003; Millar et al., 2019), dispositional favorability towards oneself that may index how much others like them (i.e., self-esteem; Leary, 1999), and people’s dispositional feeling of being left out, unsupported, not belonging (i.e., loneliness; Russell, 1996). Indeed, each trait revealed different processes of responding to dealbreakers/makers. Mate value was only sensitive to dealmakers, especially among men in the short-term. This may be evidence of the idea that in initial mate choice decisions, men are looking for reasons to say “yes” especially in short-term mating contexts (Buss and Schmitt, 1993). While mate value had no effects in relation to decisions towards dealbreakers, self-esteem was associated with responses to both dealbreaker and dealmaker information. It appears that men, more than women, with high self-esteem felt empowered to accept desirable partners and reject undesirable ones more than those with less self-esteem. With less bargaining power in the dating market on average, men with a greater sense of social inclusion may know they have a good chance to find other mates—or at least not to be alone—which enables them to be “choosier” in the context of romantic and sexual relationships. And last, loneliness was especially sensitive to dealbreaker and dealmaker responses with lonelier people saying “no” to dealbreakers less and “no” to dealmakers more. This suggests a paradox of mate choice for those who are lonely. They may be accepting lower quality partners, which could lead to more loneliness because of the eventual relationship termination created by lowering one’s standards in their partners while rejecting the very partners who they might be able to have a sustained relationship with thereby decreasing their loneliness. That is, lonely people may unfortunately engage in decision-making that perpetuates their social isolation.

## Limitations and conclusion

Despite being experimental, well-powered, more ecologically valid than prior research, and systematic in its attempts to understand individual differences in mating decisions, our study was limited in several ways. First, we relied only on W.E.I.R.D. participants (Henrich et al., 2010) from MTurk to collect participants (during COVID-19), therefore future research should attempt to understand the decision-making processes in initial courtship moments in various countries/cultures. Second, we hoped that the three-dimensional measure of mate value we used would bear aspect-level effects, but we failed to find much differentiation in our results on the three subsets of mate value. Third, while experimental in the context and content of information learned, the role of individual differences like self-esteem can only be treated as cross-sectional because we failed to manipulate it, e.g., with a bogus feedback or priming method. Fourth, a key reason dealbreakers are worth investigating is that they may be given a heavier weighting because loss aversion is stronger than gain approach (Kahneman and Tversky, 1979; Jonason et al., 2015) and the way people balance these costs and benefits may be captured by personality traits like those we included here and likely others (such as attachment, love styles, or pathological personality traits). Fifth, our measure of dealbreakers/makers are rather *ad hoc* and could be composed of other items or analyses could zoom in on item-level effects, both of which might reveal different/interesting effects. By focusing on composite measures, we hoped to minimize idiosyncrasies and Type 1 error.

When people are in the initial stages of courtship, they are being naïve researchers, collecting data, building hypotheses, and making decisions about whether they want to continue the relationship with their new, potential partner. There are two kinds of information people can learn, some of which should increase the appeal of the potential partner whereas others should decrease it. These dealmakers and dealbreakers feed into different decision-making processes, one geared towards avoiding mating mistakes and the other seeking high quality partners. In this study, we replicated and extended previous research on this holistic process of decision-making in courtship. Dealbreakers loomed larger than dealmakers in mate choice, as would be expected from prospect theory, and it was women who they loomed heavier for, consistent with sexual strategies theory. Individual differences in social inclusion were associated with different patterns in decision-making, suggesting that people with more self-esteem and mate value (men in particular) may calibrate their mating choices based on the assumption that they have high value, whereas lonely people may be perpetuating their loneliness with their decisions in response to dealbreakers and dealmakers.

## Data availability statement

The datasets presented in this study can be found in online repositories. The names of the repository/repositories and accession number (s) can be found at: https://osf.io/anw5k/.

## Ethics statement

The studies involving human participants were reviewed and approved by Western Sydney University (H14099) and Putnam Valley High School (72021). The patients/participants provided their written informed consent to participate in this study.

## Author contributions

PJ oversaw all aspects of this study and is responsible for the design, analyses, and the conclusions. KW assisted PJ in the analyses and writing. AL collected the data and reviewed a draft. LA assisted with the study design and writing. All authors contributed to the article and approved the submitted version.

## Funding

PJ was partially funded by the Polish National Agency for Academic Exchange (PPN/ULM/2019/1/00019/U/00001) and a grant from the National Science Center of Poland (2019/35/B/HS6/00682).

## Conflict of interest

The authors declare that the research was conducted in the absence of any commercial or financial relationships that could be construed as a potential conflict of interest.

## Publisher’s note

All claims expressed in this article are solely those of the authors and do not necessarily represent those of their affiliated organizations, or those of the publisher, the editors and the reviewers. Any product that may be evaluated in this article, or claim that may be made by its manufacturer, is not guaranteed or endorsed by the publisher.

## Footnotes

^1^There was no difference in overall responses between participant in (*M* = 0.73, *SD* = 1.62) or not in (*M* = 0.26, *SD* = 1.90) a committed relationships (*t* = −1.81, *p* = 0.07) or between those who identified as heterosexual (*M* = 0.49, *SD* = 1.75) and homosexual (*M* = 1.06, *SD* = 0.81; *t* = −1.07, *p* = 0.29).

^2^There was no difference in overall responses between participants in (*M* = 21.76, *SD* = 4.68) and not in (*M* = 21.28, *SD* = 5.07) a committed relationship (*t* = 0.78, *p* = 0.43) or between participants who identified as homosexual (*M* = 21.17, *SD* = 7.09) or heterosexual (*M* = 21.64, *SD* = 4.63; *t* = 0.34, *p* = 0.74).

^3^https://osf.io/anw5k/ (accessed September 04, 2022).

^4^Study 2 used different wording than Study 1 and Jonason et al. (2020b) for the dealbreaker regarding alcohol consumption. The item used in Study 2 was accidentally changed from "Drinks quite a bit" to "Drinks a lot of alcohol."

^5^Long-term attractiveness was positively correlated with short-term attractiveness (*r* = 0.54, *p* < 0.001) and unattractiveness (*r* = 0.23, *p* < 0.001) and short-term attractiveness was positively correlated with unattractiveness (*r* = 0.50, *p* < 0.001).

^6^The average dealbreaker rating was less than the mid-point on the scale of zero (*t*[152] = −2.52, *p* = 0.013).

^7^The average dealbreaker count of "yes’s" was more than zero (*t*[303] = 17.98, *p* < 0.001).
